# Supplementary material for: Host dietary specialization and neutral assembly shape gut bacterial communities of wild dragonflies
Source: PeerJ. 2019 Nov 18;7:e8058. doi: 10.7717/peerj.8058 (PMC6870522; doi:10.7717/peerj.8058)
Supplement: Supplemental Information 1 [file peerj-07-8058-s001.docx]

**SUPPLEMENTARY FIGURES**

**Fig S1:** QIIME analysis flowchart.


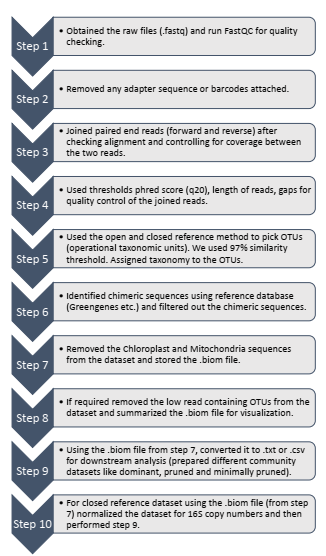


**Fig S2.** Boxplot showing read counts (library size) for gut-bacterial and gut content MiSeq samples (after quality trimming and pair-joining).


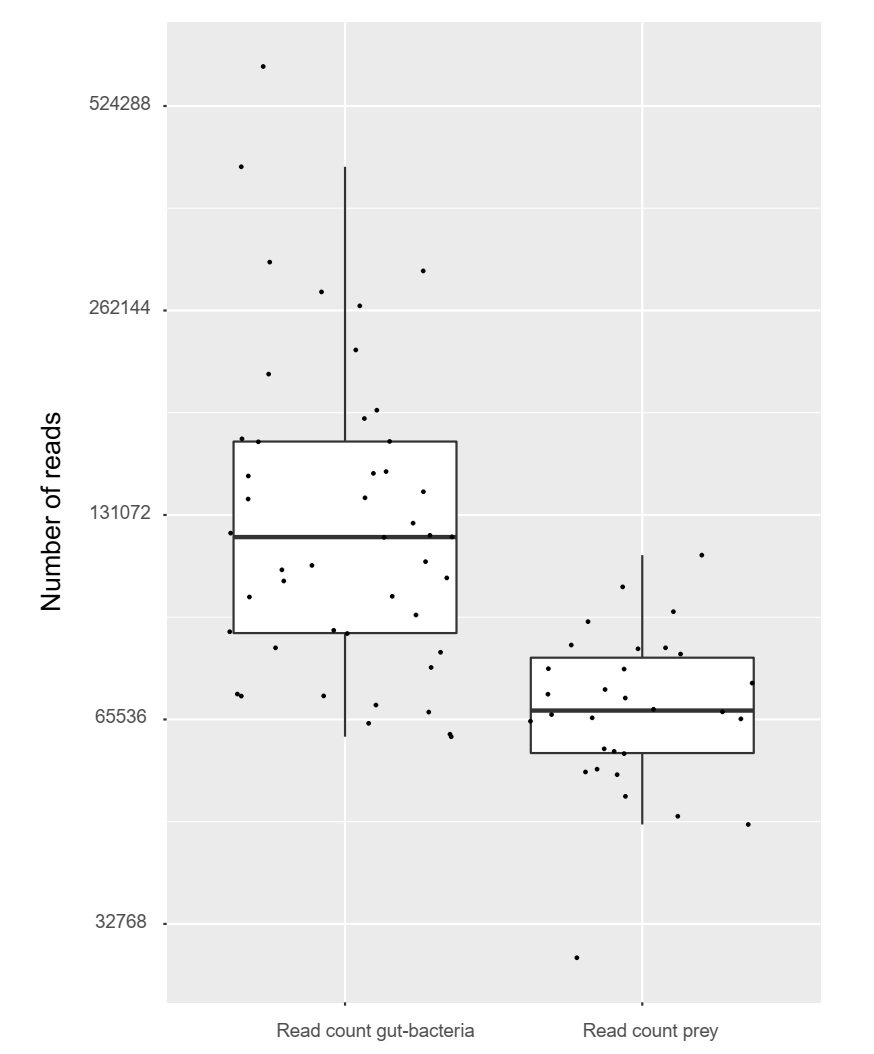


**Fig S3:** Boxplot showing read counts for prey and host after OTU clustering and taxonomic assignment. 12.4% (average) out of 2.1 million reads were prey reads across all the samples.


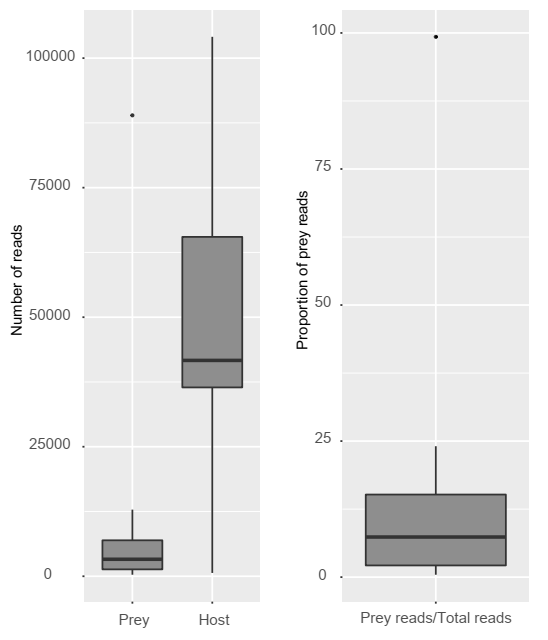


­**Fig S4.** Rarefaction plot showing a change in Faith’s phylogenetic diversity (PD) as a function of sampling depth (number of reads per dragonfly sample). All samples (N=47) tend to saturate by a depth of 2400 reads/sample. Grey circles show the median diversity for 100 iterations of sampling reads at each sampling depth. These points are summarized by boxplots showing median phylogenetic diversity at each sampling depth; large circles are outliers. Red points indicate the dragonfly sample that was removed from the dataset since it had a low sampling depth.


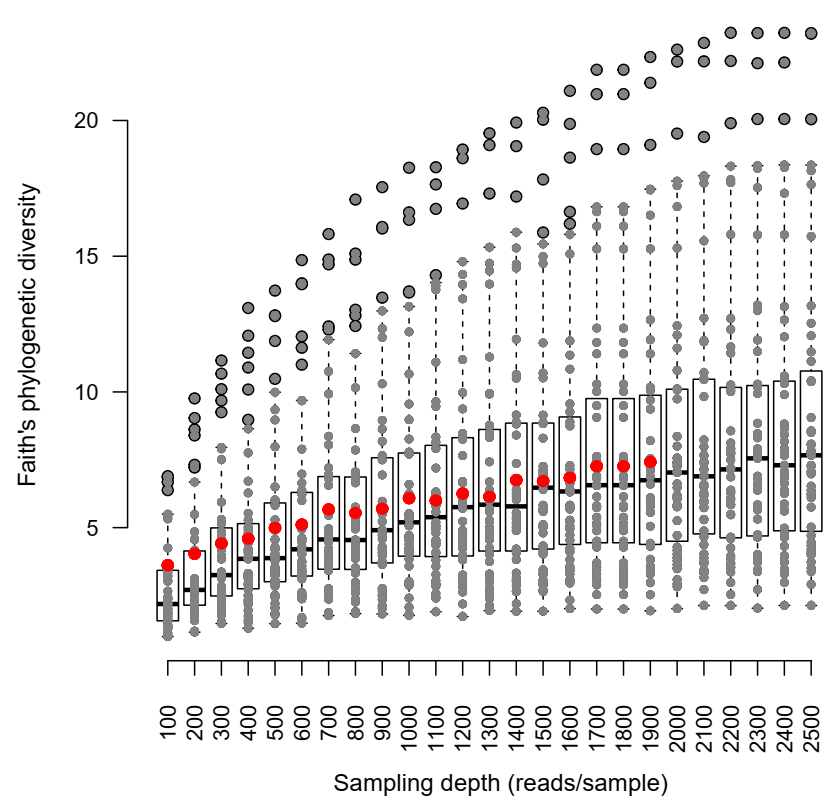


**Fig S5.** Capdiscrim plots based on linear discriminant analysis, showing clustering of the host dragonflies based on the dominant gut bacterial community composition (open referenced).


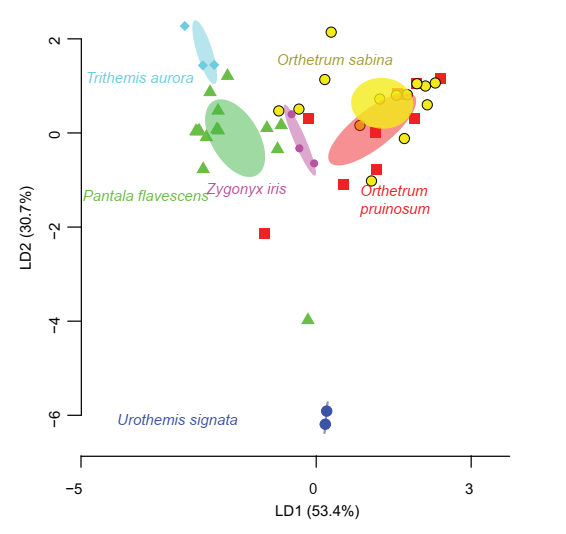


**Fig S6.** Capdiscrim plots based on linear discriminant analysis, showing clustering of (A) the host dragonflies (main 3 species), (B) geographic locations from which the samples were collected based on the dominant gut bacterial community composition (closed reference).


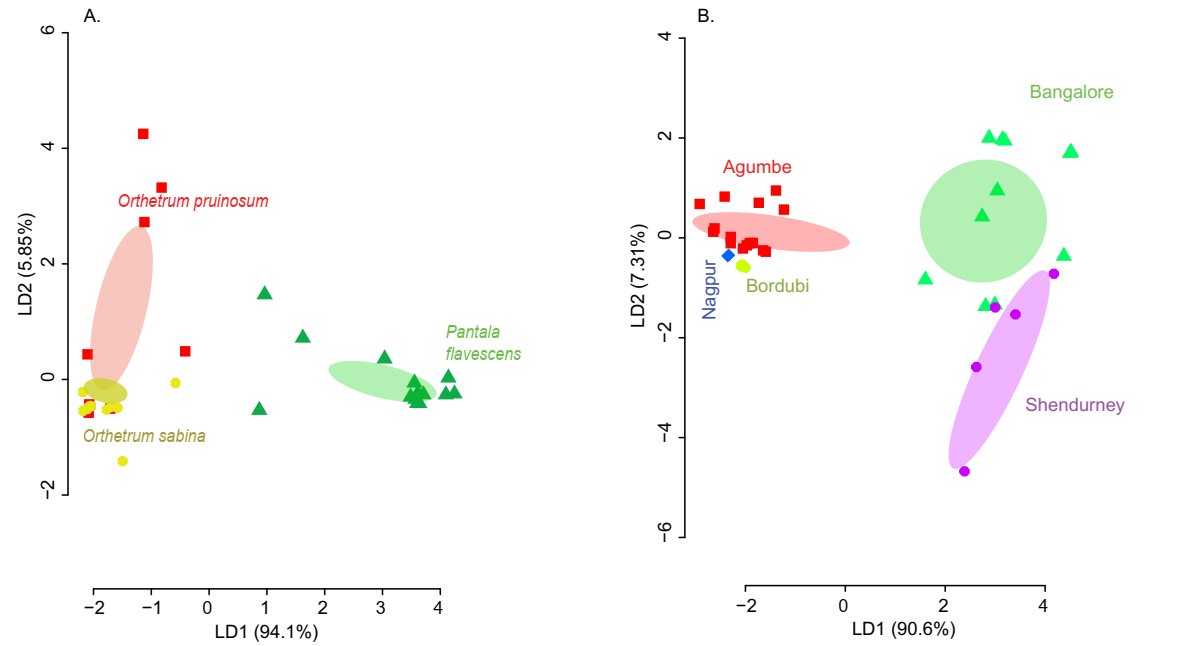


**Fig S7.** Linear discriminant (LD) plots showing two dominant linear discriminants (LD) that group dragonfly samples based on their mean rarefied gut bacterial community composition (based on Bray-Curtis distance and open reference OTU picking). Percentage of variance explained by each LD is indicated in parentheses. Each point represents a host individual. Ellipsoids represent 95% confidence intervals around each group mean, calculated from LD values. Clustering of dragonfly samples based on (A) host species identity (3 well-sampled species) (Table S1A), (B) sampling location (for 3 well-sampled species) (Table S1A). (C) Clustering of 5 dragonfly species from Shendurney (Table S1), based on their gut bacterial composition.


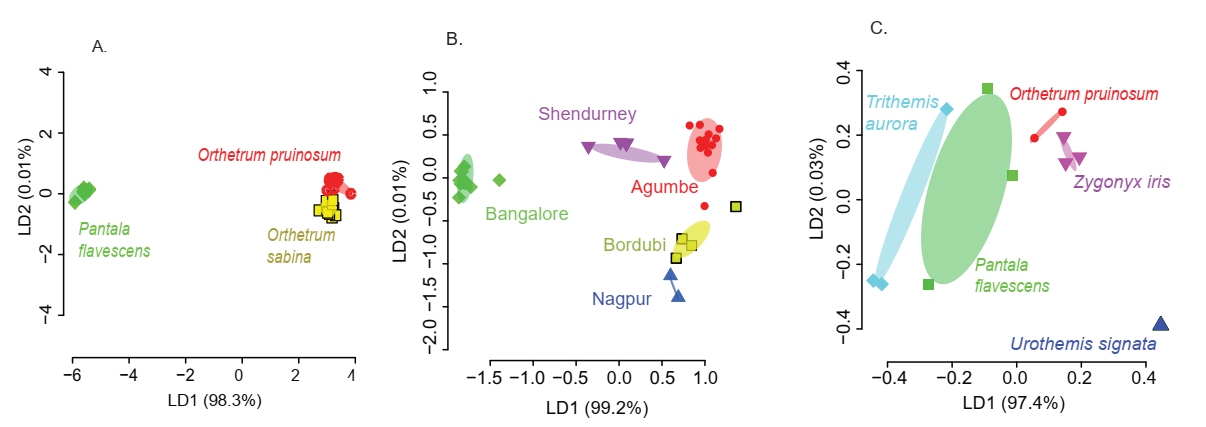


**Fig S8.** (A-B) α diversity (Shannon’s index) of the pruned gut bacterial communities across (A) host species (*Orthetrum sabina*, OP- *Orthetrum pruinosum*, PF- *Pantala flavescens*, TA-*Trithemis aurora*, US- *Urothemis signata,* ZI – *Zygonyx iris*; well-sampled dragonfly species are highlighted by the grey region), and (B) sampling location (AG- Agumbe, BD- Bordubi, BN- Bangalore, NG- Nagpur and SS- Shendurney. Five different species of dragonflies were sampled only in Shendurney). (C-D) β_w_ diversity across (C) host species (the 3 well-sampled dragonflies are highlighted by the grey region) and (D) sampling location. (E-F) Venn diagrams showing shared vs unique OTUs across (E) host species (*Urothemis signata* with 2 unique OTUs is not shown because a 6-way Venn diagram made it difficult to discern patterns) and (F) sampling location.


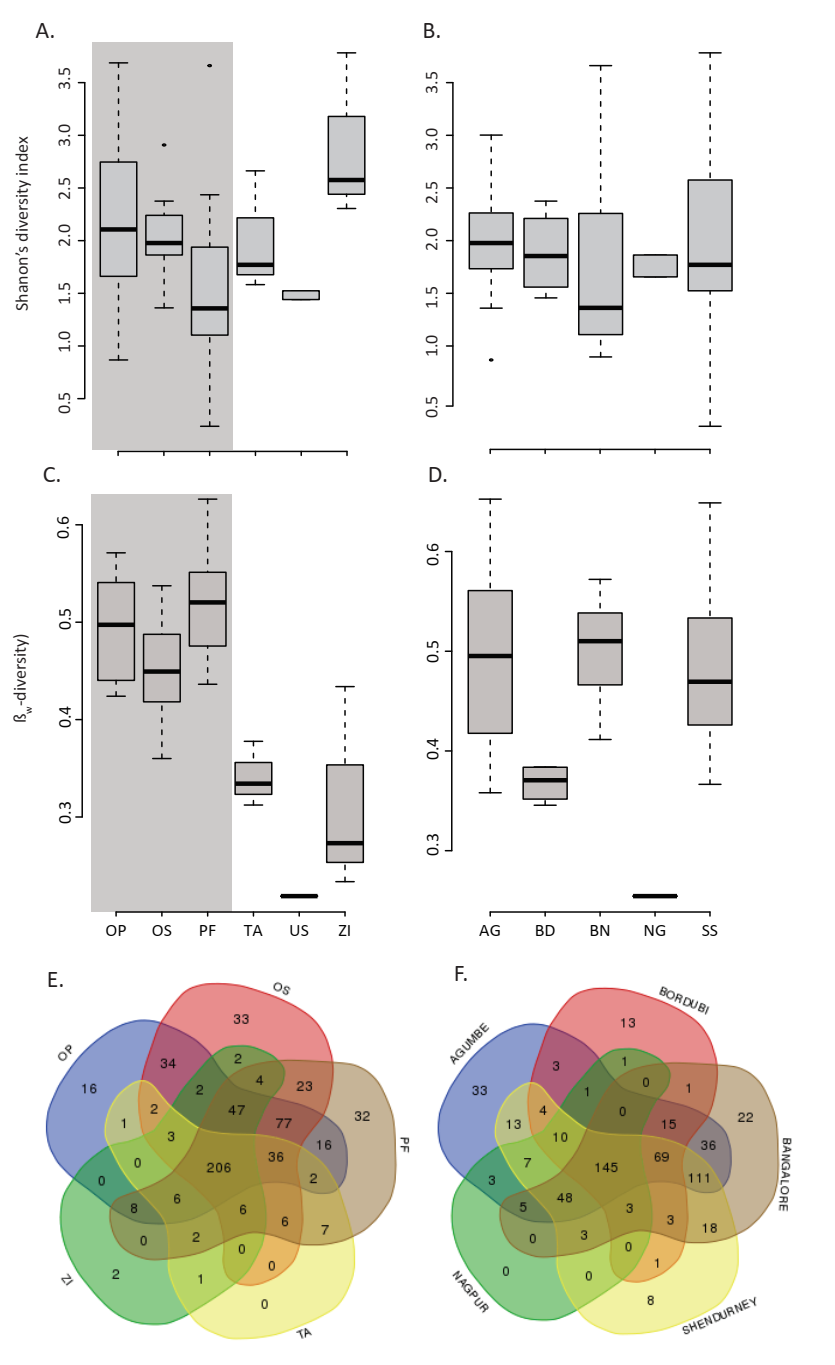


**Fig S9.** (A-B) α diversity (Shannon’s index) of mean rarefied pruned gut bacterial communities across (A) host species (*Orthetrum sabina*, OP- *Orthetrum pruinosum*, PF- *Pantala flavescens*, TA-*Trithemis aurora*, US- *Urothemis signata,* ZI – *Zygonyx iris*), and (B) sampling location (AG- Agumbe, BD- Bordubi, BN- Bangalore, NG- Nagpur and SS- Shendurney). Five different species of dragonflies were sampled only in Shendurney. (C-D) α diversity of mean+1SD rarefied community across (C) host species and (D) sampling location. (E-F) α diversity of mean–1SD rarefied community across (E) host species and (F) sampling location.


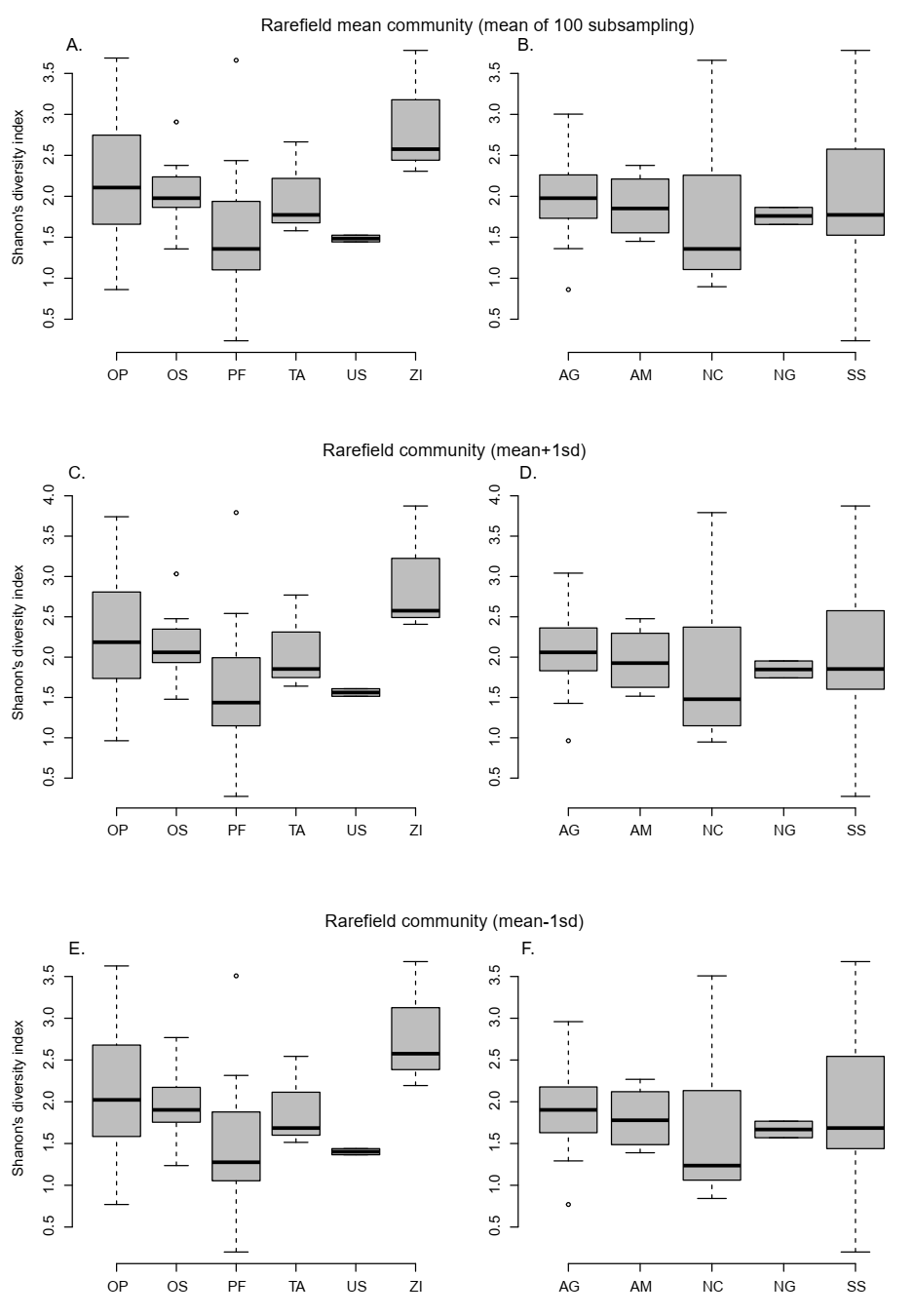


**Fig S10:** (A-B) β diversity (Aitchison’s index) of pruned gut bacterial communities across (A) host species (*Orthetrum sabina*, OP- *Orthetrum pruinosum*, PF- *Pantala flavescens*, TA-*Trithemis aurora*, US- *Urothemis signata,* ZI – *Zygonyx iris*), and (B) sampling location (AG- Agumbe, BD- Bordubi, BN- Bangalore, NG- Nagpur and SS- Shendurney). Five different species of dragonflies were sampled only in Shendurney.


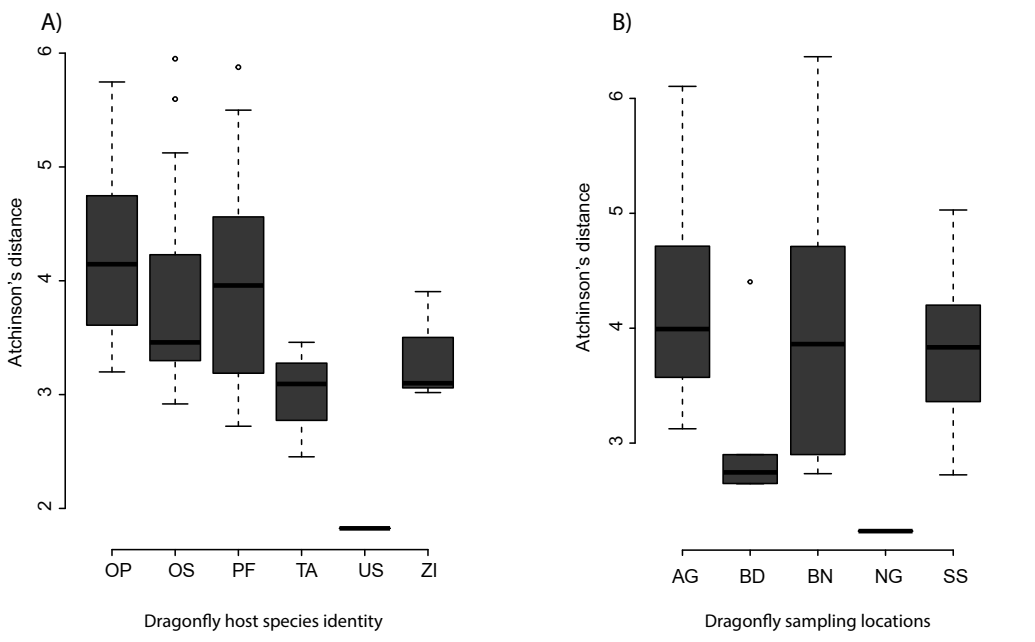


**Fig S11.** (A-B) β_w_ diversity of the mean rarefied pruned gut bacterial communities across (A) host species (*Orthetrum sabina*, OP- *Orthetrum pruinosum*, PF- *Pantala flavescens*, TA-*Trithemis aurora*, US- *Urothemis signata,* ZI – *Zygonyx iris*), and (B) sampling location (AG- Agumbe, BD- Bordubi, BN- Bangalore, NG- Nagpur and SS- Shendurney). Five different species of dragonflies were sampled only in Shendurney. (C-D) β_w_ diversity of mean+1SD rarefied community across (C) host species and (D) sampling. (E-F) β_w_ diversity of mean–1SD rarefied community across (C) host species and (D) sampling location.


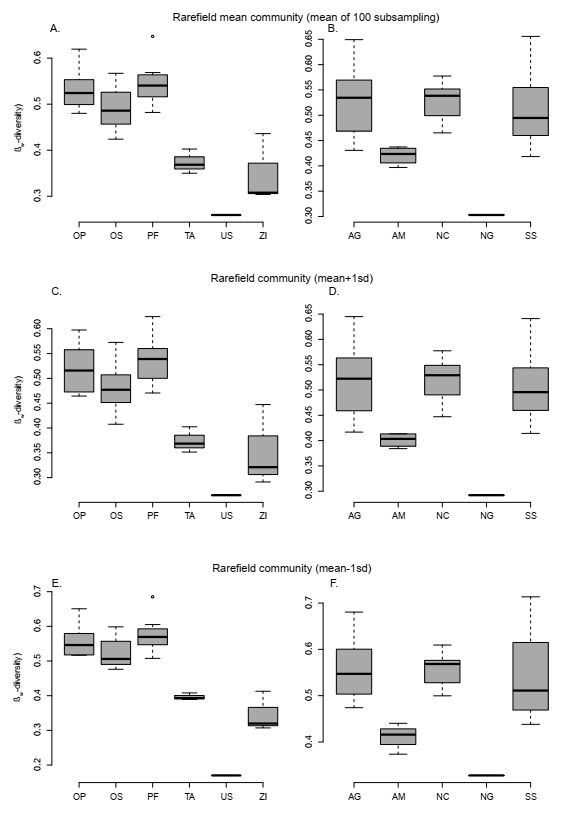


**Fig S12.** Capdiscrim plot (LD) showing clustering of dominant bacterial communities based on their host identity, and sampling location after omitting OTUs from the family (A-B) Rickettsiaceae, and (C-D) Enterobacteriaceae.


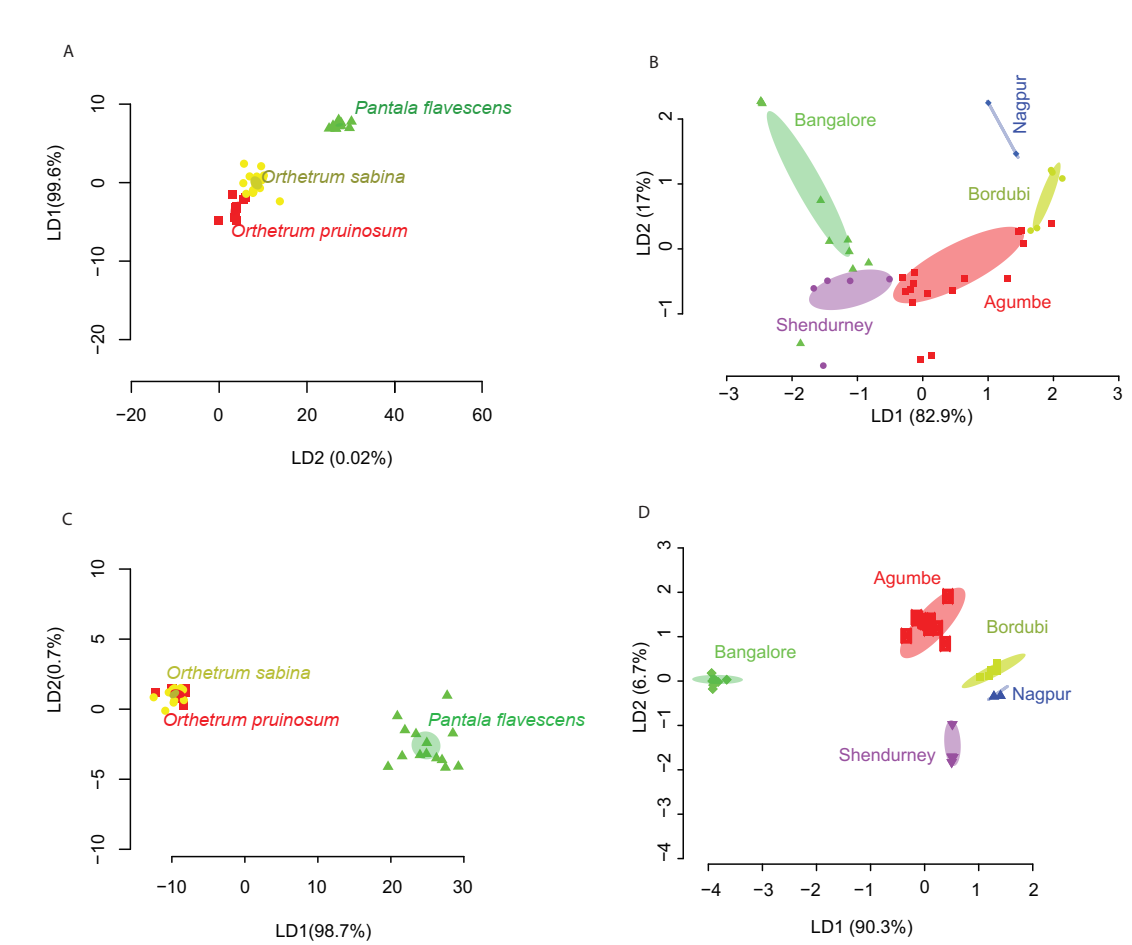


**Fig S13.** Abundance of (A-B) Rickettsiaceae, and (C) Enterobacteriaceae, in the dominant gut bacterial communities compared across (A, C) host species, and (B, D) location. For this analysis, only the three well-sampled dragonflies were considered (Table S1A).


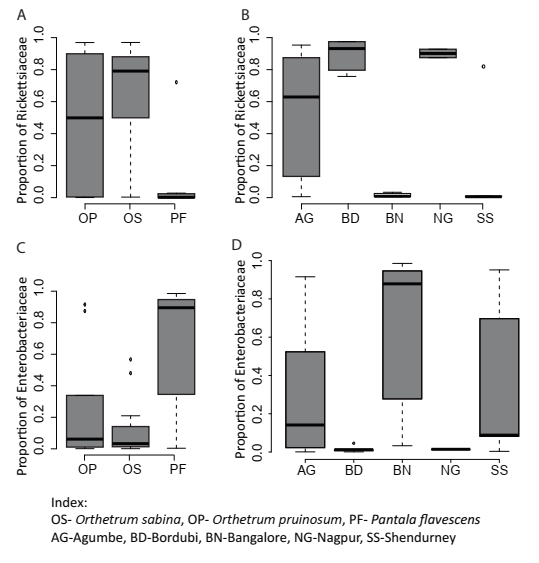


**Fig S14.** The ratio of internal control and *Wolbachia* primer C_T_ values in three dragonfly species.


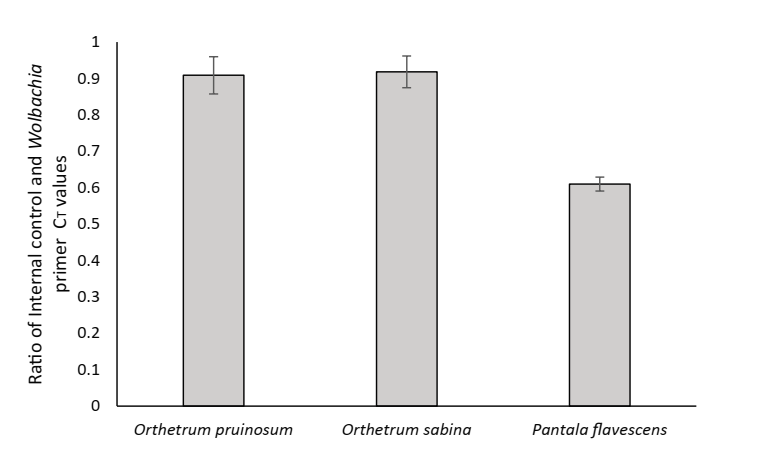


**Fig S15.** OTU richness of the pruned bacterial community across (A) dragonfly host species (the grey box marks 3 well-sampled host species; rest of the samples were collected from Shendurney, Table S1A-B), and (B) sampling location (5 dragonfly species were collected from Shendurney, Table S1A-B).


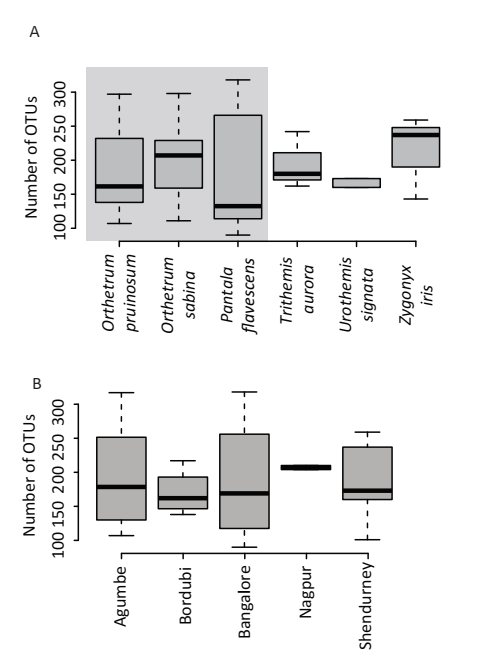


**Fig S16.** Boxplots show (A) Shannon diversity and (B) OTU richness of bacterial and prey communities of three dragonfly species, using mean rarefied open reference communities*.* Asterisks indicate significant differences in richness (Kruskal Wallis test). (C) Clustering of dragonfly samples based on dietary composition using LD analysis (as described in Fig S7) for mean rarefied open reference community.


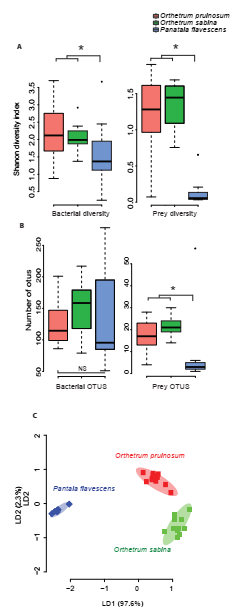


**Fig S17.** Composition of gut contents of (A) *P. flavescens* (B) *O. pruinosum* and (C) *O. sabina*. The x-axis shows major insect orders identified in the diet. The left y-axis (grey bars) shows the proportion of reads contributed by each insect order; the right-hand y-axis (blue dots) shows the number of OTUs contributed by each insect order.


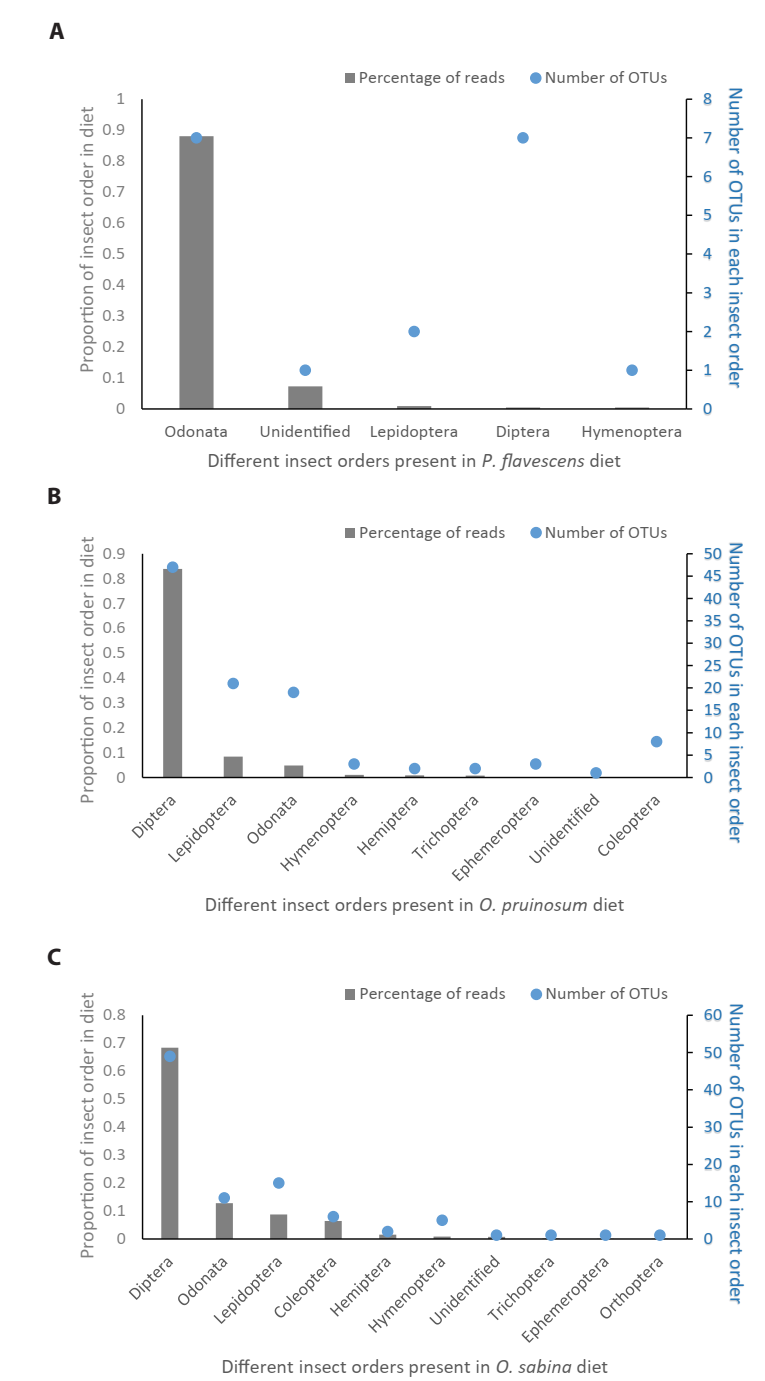


**Fig S18**. Capdiscrim plot based on linear discriminant analysis, showing clustering of the host dragonflies (main 3 species sampled in Agumbe region) based on the dominant gut bacterial community composition (open referenced OUT picking). The clustering pattern observed here is similar to that observed based on host identity (for dragonflies collected from different locations; Fig 2A) and based on prey community composition (for dragonflies collected from Agumbe region; Fig 3C).


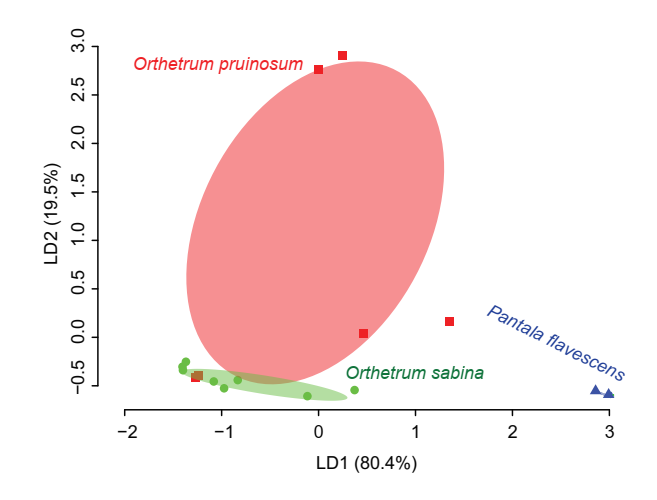


**Fig S19**. Plots showing Sloan’s neutral model fit, for OTU occurrence vs abundance obtained by pooling all dragonfly species for each location. Each dot represents a single bacterial OTU. Occurrence frequency indicates the number of hosts in which a particular OTU appears. The red line represents the neutral model fit, with 99% CI marked in orange. The R^2^ value of the fit is given in each plot.


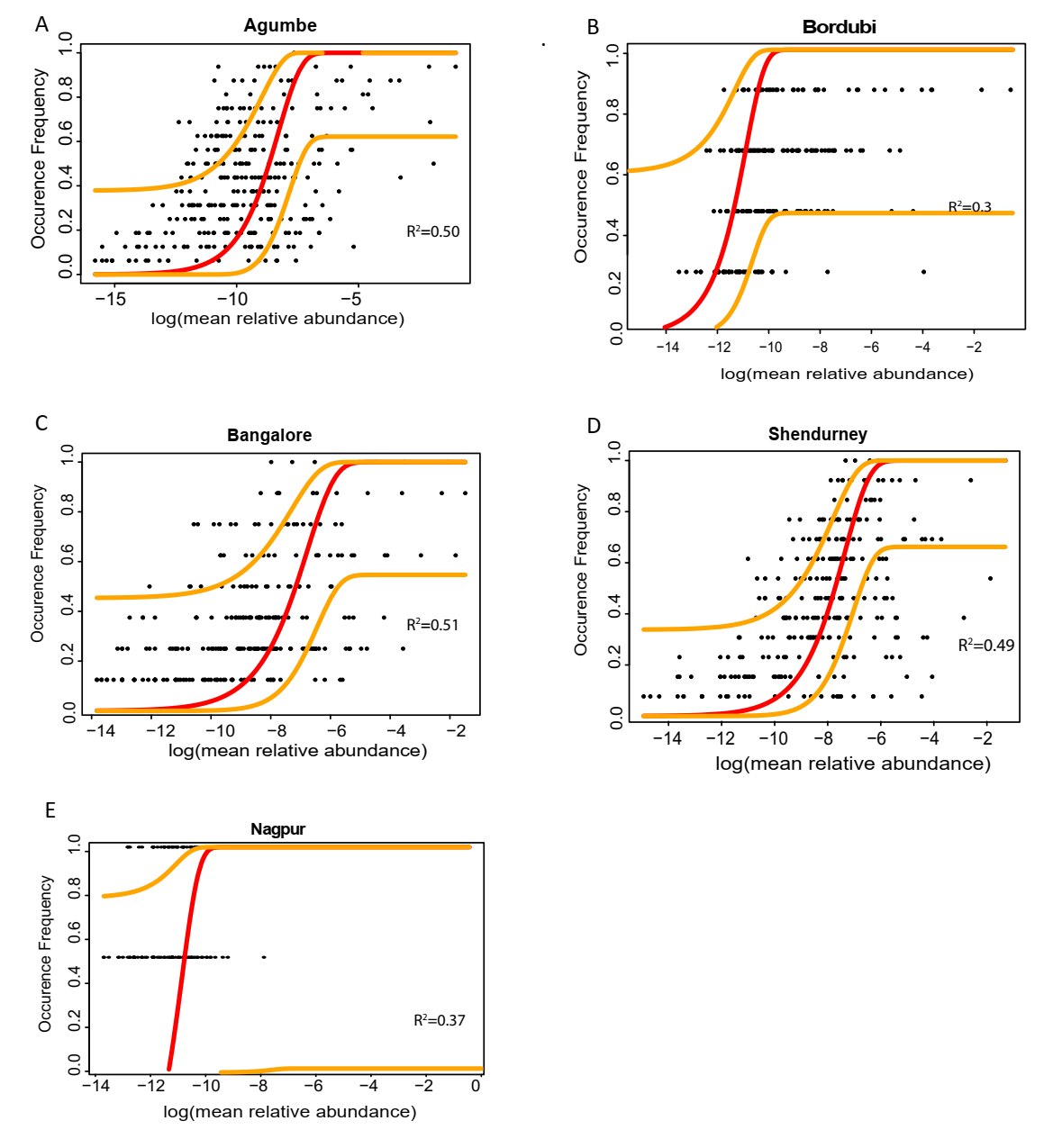


**Fig S20**. Boxplots showing the taxonomic diversity of bacteria predicted to be neutrally assembled or facing positive or negative selection, across geographic locations.


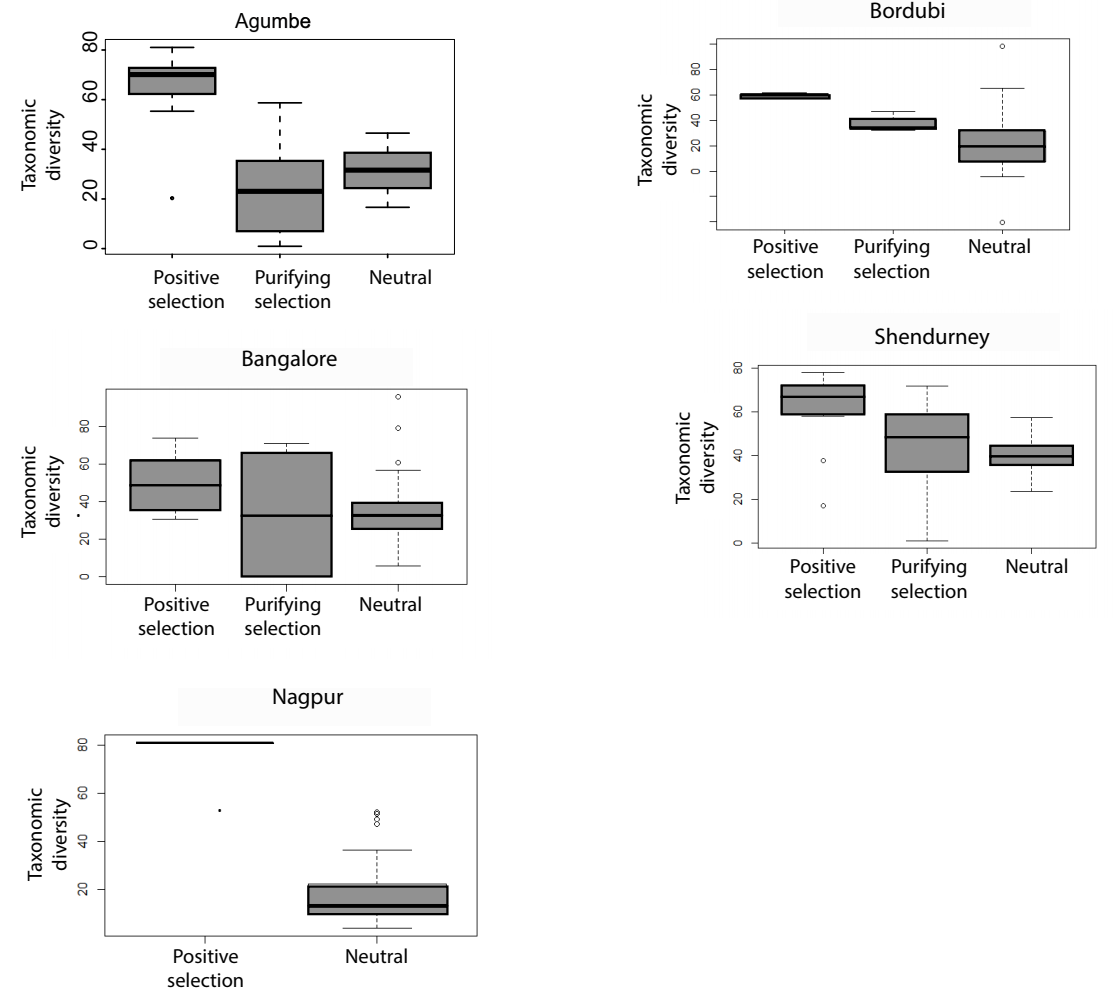


**Fig S21.** Boxplots showing the proportion of bacteria predicted to be (A-B) neutrally assembled vs (D-F) under positive selection, across (A, C) host species (B, D) locations. (OS- *Orthetrum sabina*, OP- *Orthetrum pruinosum*, PF- *Pantala flavescens*, TA-*Trithemis aurora*, ZI – *Zygonyx iris*). Data for *Urothemis signata* are not shown because we had low sample sizes for this species.


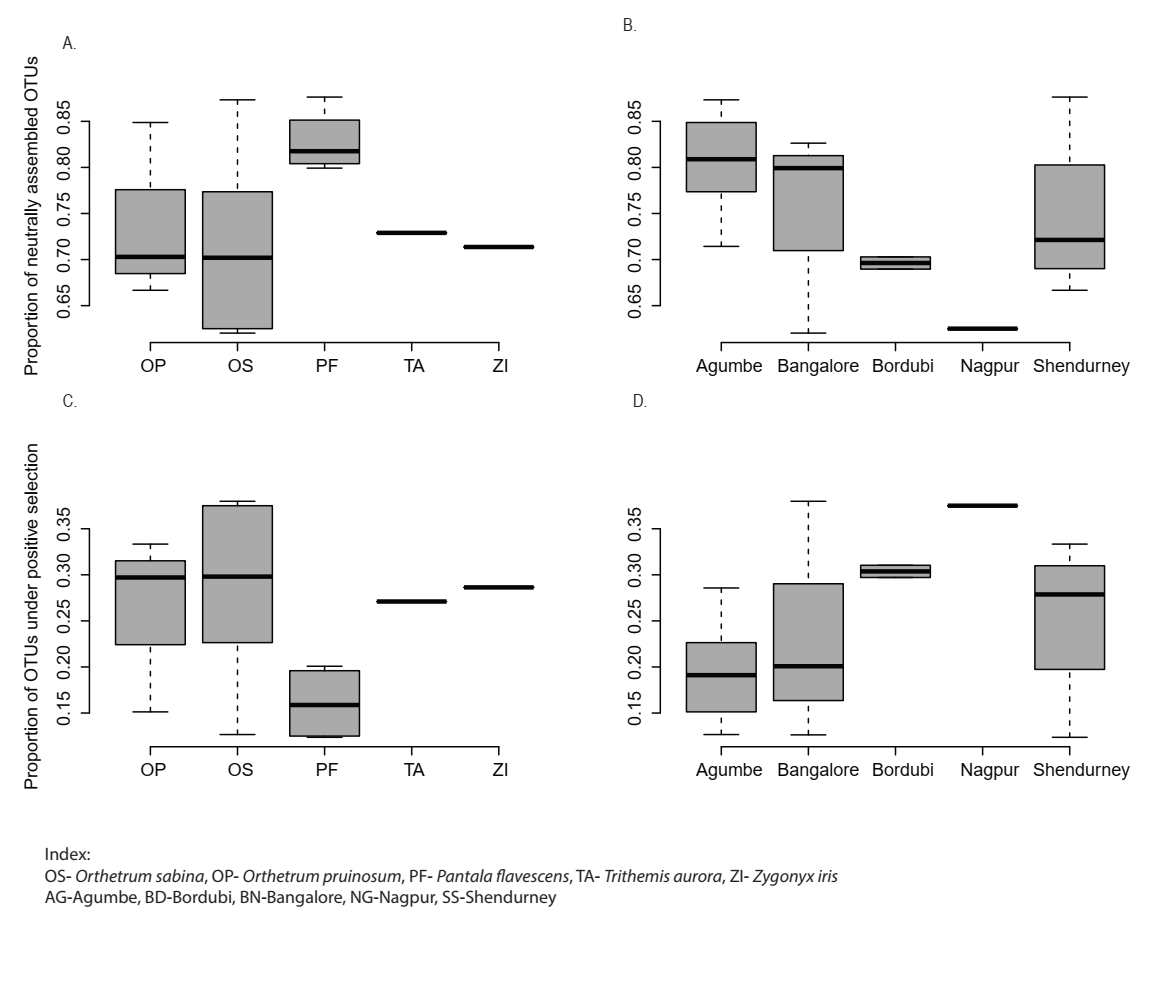


**Fig S22.** Confocal microscopy images showing *Bacillus thuringiensis* cells on a heat-fixed smear (60X oil immersion optical zoom) stained with (A) DAPI, and (B) eubacterial probe (identical field of views).


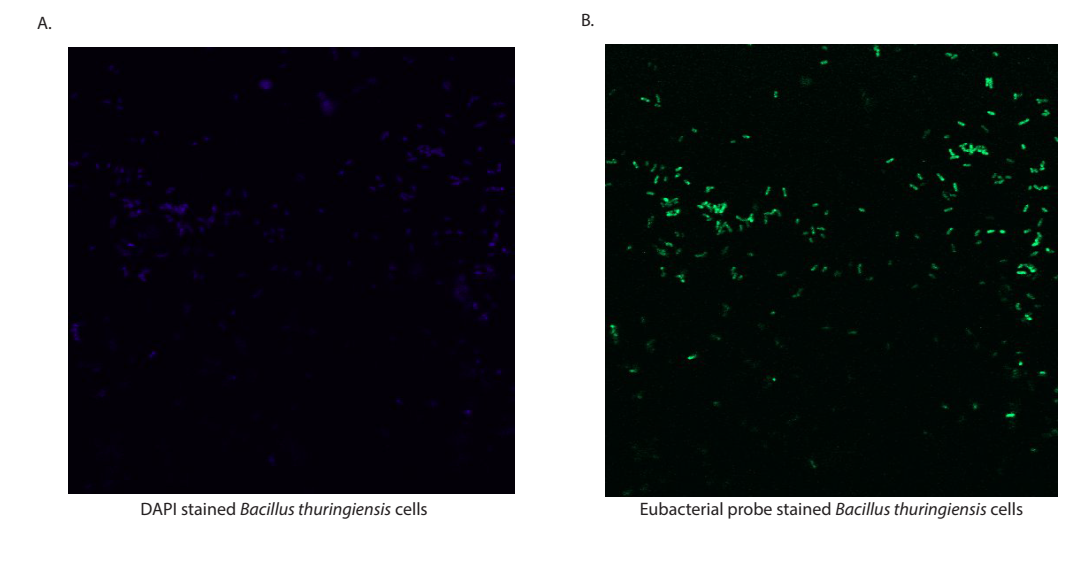


**SUPPLEMENTARY TABLES**

**Table S1:** Number of dragonflies sampled for a given species, and location. The two species of *Orthetrum* use similar foraging habitats near water bodies, with micro-habitat separation. *O. pruinosum* forages in the interior part of water bodies, with rare visits to land. *O. sabina* generally forages around the land near the water bodies. *P. flavescens* forages 3-4 meters in the air, and rarely settles down; it is not restricted to water bodies. Except for *Zygonyx iris* which has a limited range (southern tip of India), all other species are found across India. All samples were collected during the monsoon/ post-monsoon season, except 3 individuals of *Orthetrum sabina* that were collected during summer from Agumbe region to examine their gut bacterial profile.

| **A) Gut bacterial community** | | | | | |
| --- | --- | --- | --- | --- | --- |
| **Species** | **Sampling Location** | | | | |
|  | **Agumbe** | **Shendurney** | **Bangalore** | **Bordubi** | **Nagpur** |
| *Orthetrum pruinosum (Crimson-tailed Marsh Hawk)* | 6 | 2 |  | 2 |  |
| *Orthetrum sabina* (Green Marsh Hawk) | 8 |  | 2 | 3 | 2 |
| *Pantala flavescens* (Globe Skimmer) | 2 | 3 | 9 |  |  |
| **Dragonfly diet diversity** |  |  |  |  |  |
| **Species** | **Sampling Location** |  |  |  |  |
|  | **Agumbe** |  |  |  |  |
| *Orthetrum pruinosum (Crimson-tailed Marsh Hawk)* | 11 |  |  |  |  |
| *Orthetrum sabina* (Green Marsh Hawk) | 10 |  |  |  |  |
| *Pantala flavescens* (Globe Skimmer) | 7 |  |  |  |  |
| **Dragonfly gut bacteria localization through FISH** | |  |  |  |  |
| **Species** | **Sampling Location** |  |  |  |  |
|  | **Bangalore** |  |  |  |  |
| *Orthetrum pruinosum (Crimson-tailed Marsh Hawk)* | 5 |  |  |  |  |
| *Orthetrum sabina* (Green Marsh Hawk) | 5 |  |  |  |  |
| *Pantala flavescens* (Globe Skimmer) | 5 |  |  |  |  |
| **qPCR to estimate the dragonfly gut bacterial load** | |  |  |  |  |
| **Species** | **Sampling Location** |  |  |  |  |
|  | **Agumbe** |  |  |  |  |
| *Orthetrum pruinosum (Crimson-tailed Marsh Hawk)* | 3 |  |  |  |  |
| *Orthetrum sabina* (Green Marsh Hawk) | 3 |  |  |  |  |
| *Pantala flavescens* (Globe Skimmer) | 3 |  |  |  |  |

| **B) Other dragonfly host species sampled for gut bacterial diversity** | |
| --- | --- |
| **Species** | **Sampling Location** |
|  | **Shendurney** |
| *Trithemis aurora* (Crimson Marsh Glider) | 3 |
| *Urothemis signata* (Greater Crimson Glider) | 2 |
| *Zygonyx iris* (Iridescent stream glider) | 3 |

**Table S2:** Effect of host species, location, and library size on the composition of the gut bacterial community (A, B) and prey community (C,D), for (A) Dominant Community (open reference, original library size), (B) Dominant Community (open reference, using post OTU assigned library size), and (C) Prey Community (open reference, original library size), and D) Prey Community (open reference, using post OTU assigned library size).

| A) | Df | SSq. | Mean SSq. | F stat | R2 | P |
| --- | --- | --- | --- | --- | --- | --- |
| Species | 2 | 1.33 | 0.66 | 2.34 | 0.09 | 0.006 |
| Location | 4 | 3.59 | 0.89 | 3.17 | 0.25 | 9.9X10-5 |
| Interaction (Species, Location) | 3 | 1.42 | 0.47 | 1.66 | 0.10 | 0.034 |
| Library size | 1 | 0.34 | 0.34 | 1.21 | 0.02 | 0.27 |
| Residuals | 27 | 7.65 | 0.28 |  | 0.53 |  |
| Total | 37 | 14.34 |  |  | 1 |  |
| B) | Df | SSq. | Mean SSq. | F stat | R^2^ | P |
| Species | 2 | 1.31 | 0.65 | 2.36 | 0.09 | 0.007 |
| Location | 4 | 3.77 | 0.94 | 3.39 | 0.26 | 9.9X10^-5^ |
| Interaction (Species, Location) | 3 | 1.39 | 0.47 | 1.68 | 0.10 | 0.034 |
| Library size | 1 | 0.26 | 0.26 | 0.95 | 0.02 | 0.44 |
| Residuals | 28 | 7.77 | 0.28 |  | 0.54 |  |
| Total | 38 | 14.52 |  |  | 1 |  |
| C) | Df | SSq. | Mean SSq. | F stat | R2 | P |
| Species | 2 | 3.24 | 1.62 | 8.11 | 0.39 | 9.9X10^-5^ |
| Library size | 1 | 0.19 | 0.19 | 0.99 | 0.02 | 0.39 |
| Residuals | 24 | 4.79 | 0.19 |  | 0.58 |  |
| Total | 27 | 8.23 |  |  | 1 |  |
| D) | Df | SSq. | Mean SSq. | F stat | R2 | P |
| Species | 2 | 3.24 | 1.62 | 8.42 | 0.39 | 9.9X10^-5^ |
| Library size | 1 | 0.37 | 0.37 | 1.95 | 0.05 | 0.07 |
| Residuals | 24 | 4.61 | 0.19 |  | 0.56 |  |
| Total | 27 | 8.23 |  |  | 1 |  |

**Table S3**. Effect of host species and location on the composition of the gut bacterial community, for (A) Dominant Community (closed reference) (B) Pruned Community (open reference), and (C) Pruned Community (closed reference).

| A) | Df | SSq. | Mean SSq. | F stat | R2 | P |
| --- | --- | --- | --- | --- | --- | --- |
| Species | 2 | 1.42 | 0.71 | 2.91 | 0.10 | 0.003 |
| Location | 4 | 3.86 | 0.97 | 3.98 | 0.28 | 9.9X10-5 |
| Interaction (Species, Location) | 3 | 1.48 | 0.49 | 2.02 | 0.11 | 0.013 |
| Residuals | 29 | 7.08 | 0.24 |  | 0.51 |  |
| Total | 38 | 13.87 |  |  | 1 |  |
| B) | Df | SSq. | Mean SSq. | F stat | R^2^ | *P* |
| Species | 2 | 1.31 | 0.65 | 2.33 | 0.09 | 0.006 |
| Location | 4 | 3.71 | 0.93 | 3.32 | 0.26 | 9.9X10^-5^ |
| Interaction (Species, Location) | 3 | 1.41 | 0.47 | 1.69 | 0.10 | 0.031 |
| Residuals | 29 | 8.10 | 0.28 |  | 0.56 |  |
| Total | 38 | 14.52 |  |  | 1 |  |
| C) | Df | SSq. | Mean SSq. | F stat | R^2^ | *P* |
| Species | 2 | 1.45 | 0.72 | 2.84 | 0.11 | 0.002 |
| Location | 4 | 3.71 | 0.93 | 3.65 | 0.27 | 0.0002 |
| Interaction (Species, Location) | 3 | 1.47 | 0.49 | 1.92 | 0.11 | 0.015 |
| Residuals | 29 | 7.12 | 0.25 |  | 0.52 |  |
| Total | 38 | 13.75 |  |  | 1 |  |

**Table S4**. The output of CAPdiscrim analysis to test the impact of (A) host species and (B) location for the open referenced dominant community of the three well-sampled dragonflies (Fig 2A-B). (C) The output of CAPdiscrim analysis for 5 dragonfly species from Shendurney (open reference dominant community) (Fig 2C).

| A) |  |  | |  | |  | |  |  | |
| --- | --- | --- | --- | --- | --- | --- | --- | --- | --- | --- |
| Classification success | 72% | P =0.001 | |  | |  | |  |  | |
| Proportion of trace | LD1 | LD2 | |  | |  | |  |  | |
|  | 0.989 | 0.0002 | |  | |  | |  |  | |
| Manova summary: Adj.. R. squared= 0.57, F=25.99, DF= 2,36, P=1.033e-07 | | | | | | | | | | |
| B) |  | |  | |  | |  |  | |  |
| Classification success | 56% | | P =0.004 | |  | |  |  | |  |
| Proportion of trace | LD1 | | LD2 | |  | |  |  | |  |
|  | 0.992 | | 0.0001 | |  | |  |  | |  |
| Manova summary: Adj.. R. squared= 0.57, F=13.78, DF= 4,34, P=8.843e-07 | | | | | | | | | | |
| C) |  | |  | |  | |  |  | |  |
| Classification success | 61.5% | | P =0.01 | |  | |  |  | |  |
| Proportion of trace | LD1 | | LD2 | |  | |  |  | |  |
|  | 0.994 | | 0.0003 | |  | |  |  | |  |
| Manova summary: Adj.. R. squared= 0.80, F=12.9, DF= 4,8, P=0.001 | | | | | | | | | | |

**Table S5.** The output of CAPdiscrim analysis to test the impact of (A) host species and (B) location for the closed reference dominant bacterial community of the three well-sampled dragonflies. C) The output of CAPdiscrim analysis for 5 dragonfly species at location Shendurney (closed reference dominant community).

| A) | | | | | | | | | | | | |  |  |  |  |  |
| --- | --- | --- | --- | --- | --- | --- | --- | --- | --- | --- | --- | --- | --- | --- | --- | --- | --- |
| Classification  success | 72% | | | P =0.001 | | |  |  | | |  | | | |  | |  |
| Proportion  of trace | LD1 | | | LD2 | | |  |  | | |  | | | |  | |  |
|  | 0.94 | | | 0.058 | | |  |  | | |  | | | |  | |  |
| Manova | DF | | | Pillai approx | | | F | Numerator DF | | | Denominator DF | | | | P | |  |
| Host Species | 2 | | | 1.099 | | | 3.93 | 18 | | | 58 | | | | 3.63X10^-5^ | |  |
| Residuals | 36 | | |  | | |  |  | | |  | | | |  | |  |
| B) | | | | | | | | | | | | | |  |  |  |  |
| Classification  success | | 64.1% | | | P =0.002 | | |  |  | | |  | | | |  | |
| Proportion  of trace | | LD1 | | | LD2 | | | LD3 |  | | |  | | | |  | |
|  |  | 0.91 | | | 0.073 | | | 0.02 |  | | |  | | | |  | |
| Manova | | DF | | | Pillai approx | | | F | Numerator DF | | | Denominator DF | | | | P | |
| Host Species | | 4 | | | 1.37 | | | 1.67 | 36 | | | 116 | | | | 0.02 | |
| Residuals | | 34 | | |  | | |  |  | | |  | | | |  | |
| C) | | |  | | |  | |  | |  |  |  |  |  |  |  |  |
| Classification success | | | 53.8% | | | P =0.01 | |  | |  |  |  |  |  |  |  |  |
| Proportion of trace | | | LD1 | | | LD2 | |  | |  |  |  |  |  |  |  |  |
|  | | | 0.982 | | | 0.0007 | |  | |  |  |  |  |  |  |  |  |
| Manova summary: Adj.. R. squared= 0.81, F=13.5, DF= 4,8, P=0.001 | | | | | | | | | | | | | | | | |  |

**Table S6.** The effect of host species, location, and their interaction on the distribution of OTUs from the bacterial family Rickettsiaceae

|  | Df | Deviance | Residual deviance | Deviance | F | *P* |
| --- | --- | --- | --- | --- | --- | --- |
| NULL |  |  | 38 | 1780430 |  |  |
| Species | 2 | 329547 | 36 | 1450883 | 9.61 | 0.0006 |
| Location | 4 | 597181 | 32 | 853702 | 8.71 | 9.6e-05 |
| Interaction  (Species, Location) | 3 | 244711 | 29 | 608991 | 4.76 | 0.008 |

**Table S7.** The effect of host species, location, and their interaction on the distribution of OTUs from the bacterial family Enterobacteriaceae.

|  | Df | Deviance | Residual deviance | Deviance | F | *P* |
| --- | --- | --- | --- | --- | --- | --- |
| NULL |  |  | 38 | 1587873 |  |  |
| Sampling Location | 4 | 408316 | 34 | 1179557 | 3.89 | 0.02 |
| Species | 2 | 207345 | 32 | 972211 | 3.95 | 0.03 |

**Table S8.** Effect of host identity and location on (A) richness (B) α diversity and (C) β diversity of dragonfly gut bacterial communities.

| A) | Df | Deviance | Residual Df | Residual deviance | F | P |
| --- | --- | --- | --- | --- | --- | --- |
| Null |  |  | 45 | 950.66 |  |  |
| Species | 5 | 41.04 | 40 | 909.63 | 0.32 | 0.9 |
| Location | 4 | 21.61 | 36 | 888.02 | 0.21 | 0.93 |
| Interaction (Species, Location) | 3 | 64.72 | 33 | 823.29 | 0.85 | 0.48 |
| B) | Df | SSq | Mean SSq | F | P |  |
| Species | 5 | 6.25 | 1.25 | 2.67 | 0.04 |  |
| Location | 4 | 1.33 | 0.33 | 0.71 | 0.59 |  |
| Interaction (Species, Location) | 3 | 1.70 | 0.56 | 1.21 | 0.32 |  |
| Residuals | 34 | 15.94 | 0.47 |  |  |  |
| C) | Df | SSq | Mean SSq | F | R^2^ | P |
| Species | 5 | 2.25 | 0.45 | 3.05 | 0.25 | 9.9X10^-05^ |
| Location | 4 | 1.18 | 0.29 | 1.99 | 0.13 | 3X10^-04^ |
| Residuals | 37 | 5.46 | 0.15 |  | 0.61 |  |
| Total | 46 | 8.88 |  |  | 1 |  |

**Table S9:** Post-hoc Tukey’s test showing pairwise differences in Shannon diversity between host species. Significant p-values are highlighted in bold.

|  | Difference | Lower CI | Upper CI | P adjusted |
| --- | --- | --- | --- | --- |
| OS-OP | -0.23753 | -1.06733601 | 0.592274 | 0.9547864 |
| PF-OP | -0.68583 | -1.52740576 | 0.155746 | 0.1680919 |
| TA-OP | -0.21346 | -1.55148423 | 1.124557 | 0.9967222 |
| US-OP | -0.73625 | -2.31069189 | 0.838197 | 0.7281907 |
| ZI-OP | 0.669241 | -0.66877903 | 2.007262 | 0.6693694 |
| PF-OS | -0.4483 | -1.20363639 | 0.307039 | 0.4932283 |
| TA-OS | 0.024067 | -1.26146129 | 1.309596 | 0.9999999 |
| US-OS | -0.49872 | -2.02880147 | 1.031368 | 0.92352 |
| ZI-OS | 0.906772 | -0.3787561 | 2.192301 | 0.3034028 |
| TA-PF | 0.472366 | -0.82079173 | 1.765524 | 0.8818731 |
| US-PF | -0.05042 | -1.58691807 | 1.486083 | 0.9999986 |
| ZI-PF | 1.355071 | 0.06191346 | 2.648229 | 0*.***0352184** |
| US-TA | -0.52278 | -2.37828423 | 1.332717 | 0.9576771 |
| ZI-TA | 0.882705 | -0.77690492 | 2.542315 | 0.6098376 |
| ZI-US | 1.405489 | -0.4500116 | 3.260989 | 0.2321671 |

**Table S10.** A) Results of a permutational analysis of variation (PERMANOVA) showing the effect of host species on the gut bacterial composition (dominant community open reference) of *Orthetrum pruinosum*, *Orthetrum sabina*, and *Pantala flavescens* collected specifically from Agumbe region*.* (B) The output of the ordination model (CAPdiscrim) testing the impact of host species on dragonfly gut bacterial community (dominant community open reference) of *Orthetrum pruinosum*, *Orthetrum sabina*, and *Pantala flavescens* collected specifically from Agumbe region.

A)

|  | Df | SSq. | Mean SSq. | F stat | R^2^ | P |
| --- | --- | --- | --- | --- | --- | --- |
| Species | 2 | 1.52 | 0.75 | 3.13 | 0.33 | 0.002 |
| Residuals | 13 | 3.15 | 0.24 |  | 0.58 |  |
| Total | 15 | 4.66 |  |  | 1 |  |

B)

| Classification success | 81% | P =0.001 |  |  |  |  |
| --- | --- | --- | --- | --- | --- | --- |
| Proportion of trace | LD1 | LD2 |  |  |  |  |
|  | 0.80 | 0.20 |  |  |  |  |
| Manova | DF | Pillai approx | F | Numerator DF | Denominator DF | P |
| Host Species | 2 | 0.96 | 5.97 | 4 | 26 | 0.002 |
| Residuals | 13 |  |  |  |  |  |

**Table S11**. R^2^ values (from non-linear least squares) and AIC values for fitted Sloan’s neutral and Binomial models.

| Location | R sq. | AIC | R sq. Binomial | AIC Binomial |
| --- | --- | --- | --- | --- |
| Agumbe | 0.5 | -1092 | 0.36 | 104 |
| Bangalore | 0.51 | -1670 | 0.41 | 105 |
| Shendurney | 0.49 | -1145 | 0.47 | 76 |
| Nagpur | 0.37 | -1247 | 0.28 | 48 |
| Bordubi | 0.3 | -1242 | 0.28 | 66 |

**Table S12.** Analysis of deviance for gut microbial assembly of individual dragonfly hosts. Effect of host species, and location on (A) proportion of neutrally assembled OTUs and B) proportion of OTUs whose distribution is consistent with positive selection.

|  | Df | Deviance | Residual deviance | Deviance | *P* |
| --- | --- | --- | --- | --- | --- |
| A) |  |  |  |  |  |
| NULL |  |  | 14 | 106.31 |  |
| Location | 4 | 32.34 | 10 | 73.96 | 1.62X10^-06^ |
| Species | 4 | 38.61 | 6 | 35.53 | 8.38X10^-08^ |
| B) |  |  |  |  |  |
| NULL |  |  | 14 | 117.45 |  |
| Location | 4 | 32.67 | 10 | 84.78 | 1.39X10^-06^ |
| Species | 4 | 43.82 | 6 | 40.96 | 6.89X10^-09^ |
